# Clinical experiences with a PEEK-based dynamic instrumentation device in lumbar spinal surgery: 2 years and no more

**DOI:** 10.1186/s13018-018-0905-z

**Published:** 2018-08-09

**Authors:** Stavros Oikonomidis, Ghazi Ashqar, Thomas Kaulhausen, Christian Herren, Jan Siewe, Rolf Sobottke

**Affiliations:** 1 Department of Orthopaedics and Trauma Surgery, Rhein-Maas Klinikum GmbH, Mauerfeldchen 25, 52146 Wuerselen, Germany; 2 Department of Orthopaedics and Trauma Surgery, University Hospital Cologne, Joseph-Stelzmann-Str. 24, 50931 Cologne, Germany; 3 Department of Trauma and Reconstructive Surgery, University Hospital RWTH Aachen, Pauwelsstraße 30, 52074 Aachen, Germany

**Keywords:** Topping off, Hybrid posterior fixation, Adjacent segment disease, Material failure, Hybrid lumbar instrumentation

## Abstract

**Background:**

Dynamic spine implants were developed to prevent adjacent segment degeneration (ASD) and adjacent segment disease (ASDi). Purpose of this study was to investigate the clinical and radiological outcomes of “topping off” devices following lumbar spinal fusion procedure using a PEEK-based dynamic rod system. Moreover, this study focused on the hypothesis that “topping off” devices can prevent ASD.

**Methods:**

This prospective nonrandomized study included patients with indication for single-level lumbar fusion and radiological signs of ASD without instability. The exclusion criteria were previous lumbar spine surgery and no sign of disc degeneration in the adjacent segment according to magnetic resonance imaging. All patients were treated with single-level lumbar interbody fusion and dynamic stabilization of the cranial adjacent segment. Patients underwent a clinical examination and radiographs preoperatively and at 1 and 2 years after surgery. Analyses were performed on clinical data collected with the German Spine Registry using the core outcome measure index (COMI) and visual analogue scale (VAS) scores for back and leg pain.

**Results:**

A total of 22 patients (6 male and 16 female) with an average age of 57.6 years were included in the study; 20 patients completed the follow-up (FU). The average COMI score was 9.0 preoperatively, 4.2 at the 1-year FU, and 4.7 at the 2-year FU. The average preoperative VAS scores for back and leg pain were 7.7 and 7.1, respectively. At the 1-year FU, the scores were 4.25 for back pain and 2.2 for leg pain, and at the 2-year FU, the scores were 4.7 for back pain and 2.3 for leg pain. At FU, failure of the dynamic topping off implant material was verified in four cases, and ASD of the segment cranial to the topping off was confirmed in three cases.

**Conclusions:**

These results demonstrate significant improvements in clinical outcomes and pain reduction after lumbar spinal fusion with topping off at 2 years after surgery. However, the implant failed due to the high rate of implant failure and the development of ASD in the segment cranial to the dynamic stabilized segment.

## Background

Lumbar and lumbosacral spinal fusion is a state of the art in lumbar spinal surgery for treating several degenerative disorders caused by changes in the lumbar spine (i.e., spinal disc herniation, lumbar spinal stenosis, and spondylolisthesis) [1, 2]. The well-known posterior rigid pedicle screw fixation system offers initial stability, a high fusion rate, and good recovery of normal sagittal parameters in the lumbar spine. Stability is restored with surgical posterior fusion using pedicle screw and rod-based systems combined with an intervertebral cage following decompression (e.g., TLIF/PLIF technique). Furthermore, this procedure has been well documented in terms of its good and excellent long-term outcomes [3].

More recent research revealed that the stiffness caused by the posterior fusion operation often results in a redistribution of stress at the neighboring level, which leads to extended mobility and increased intradiscal pressure in the adjacent segments. These biomechanical changes can lead to new complications, such as adjacent segment degeneration (ASD) accompanied by facet hypertrophy, facet arthritis, and a higher risk of adjacent segment disease (ASDi) [4–7].

The general incidence of ASD varies from 5.2 to 18.5% at 2–5 years after lumbar fusion; however, Moreau et al. reported a rate > 20% for degenerative spondylolisthesis at 2 years after lumbar fusion [8, 9]. Risk factors for the manifestation of ASD are being > 60 years old, having an increased body mass index, preexisting disc and facet joint degeneration, the length of the fusion, and decreased postoperative lumbar lordosis in the sagittal alignment [10]. In the literature, the impact of ASD on clinical outcome is unclear [10]; however, ASDi may result in new clinical symptoms that are detectable adjacent to the previously fused segment.

Dynamic spine implants were developed to prevent ASD. These devices provide dynamic stabilization of the instrumented segment and, in focus to the adjacent segment, reduce load sharing and prevent hypermobility of the adjacent segment. Biomechanical studies reported a reduced range of motion (ROM) and load sharing of the adjacent segment when dynamic stabilization devices were used [11]. Khoueir et al. classified posterior dynamic stabilization devices into three groups: (1) interspinous spacer devices, (2) pedicle screw/rod-based devices, and (3) total facet replacement systems [12]. Many dynamic spine implants have been introduced in recent years, including purely dynamic or hybrid (semi-rigid) implants; however, it is uncertain whether patients benefit from these implants. Topping off systems provide dynamic stabilization for the segment cranially adjacent to the fusion.

The aim of this study was to assess the patient-dependent clinical and radiological outcomes within a 2-year follow-up (FU) period following lumbar interbody fusion using a dynamic instrumentation device to stabilize the segment superior to the rigid instrumented segment (topping off). Furthermore, this study also focused on the development of ASD in the segment superior to the dynamic instrumented level.

## Methods

### Study design

An observational prospective nonrandomized cohort study of patients with monosegmental degenerative alteration or spondylolisthesis of the lumbar spine and an indication for lumbar fusion was conducted (Table 1). Further inclusion criteria were radiological signs of degeneration without instability in the cranially adjacent segment (Pfirrmann grade 2–4) [13]. Detailed inclusion and exclusion criteria are listed in Table 1 [14]. Diagnosis was based on clinical and radiological examinations as well as magnetic resonance imaging (MRI). All patients underwent single-level lumbar interbody fusion using the transforaminal lumbar interbody fusion (TLIF) or posterior lumbar interbody fusion (PLIF) technique and additional dynamic instrumentation (topping off) of the segment superior to the rigid instrumented level according to segment pathology (Table 2). Three senior and “Master-certified” (German Spine Society) spinal surgeons performed the operations. Indications for lumbar spinal fusion were performed based on the radiological and clinical findings, and the Modic classification was used to characterize the grade of osteochondrosis [15].

**Table 1:** Inclusion and exclusion criteria

Inclusion criteria	Exclusion criteria
• Indication for monosegmental lumbar spinal fusion with osteochondrosis (Modic grades I–III) or spondylolisthesis (Meyerding grades I–III) with instability • Radiological signs of degeneration without instability in the level cranially adjacent to the intended fusion • Definition of adjacent segment degeneration using MRI (Pfirrmann grades II–IV)	• No degeneration in the segment cranial to the segment intended for fusion • Previous lumbar surgery • Motor deficits • Scoliosis with a Cobb angle > 25° • Spondylolisthesis (Meyerding grade > III) • No prior history of metabolic bone disease • No previous osteoporotic fracture of the lumbar vertebrae

**Table 2:** Pathology of the index (fusion) and adjacent segment

Cases	Pathology of the index segment	Pathology of the adjacent segment (Pfirrmann classification)
1	Degenerative spondylolisthesis II° L4–5 and absolute LSS	L3–4: grade III
2	Erosive osteochondrosis L5–S1 Modic III° and foraminal stenosis L5 right	L4–5: grade II
3	Degenerative spondylolisthesis II° L4–5 and absolute LSS	L3–4: grade II
4	Isthmic spondylolisthesis I° L5–S1	L4–5: grade III
5	Erosive osteochondrosis L5–S1 Modic III° and absolute LSS L5–S1	L4–5: grade III
6	Degenerative spondylolisthesis II° L4–5 and erosive osteochondrosis L4–5 Modic III°	L3–4: grade III
7	Isthmic spondylolisthesis II° L5–S1	L4–5: grade III
8	Erosive osteochondrosis L5–S1 Modic III°	L4–5: grade III
9	Degenerative spondylolisthesis II° L4–5 with disc herniation	L3–4: grade II, LSS
10	Degenerative spondylolisthesis II° L4–5	L3–4: grade IV, LSS
11	Erosive osteochondrosis L4–5 Modic III° and absolute LSS	L3–4: grade III, LSS
12	Erosive osteochondrosis L4–5 Modic III° and absolute LSS	L3–4: grade IV
13	Degenerative spondylolisthesis II° L4–5 and absolute LSS	L3–4: grade III
14	Degenerative spondylolisthesis II° L4–5	L3–4: grade II, foraminal stenosis bilateral
15	Erosive osteochondrosis L4–5 Modic II° and absolute LSS	L3–4: grade II
16	Erosive osteochondrosis L5–S1 Modic III°	L4–5: grade II
17	Degenerative spondylolisthesis I° L5–S1 and absolute LSS	L4–5: grade III
18	Degenerative spondylolisthesis I° L5–S1	L4–5: grade IV, disc herniation
19	Degenerative spondylolisthesis II° L4–5 and absolute LSS	L3–4: grade III
20	Erosive osteochondrosis L5–S1 Modic III° and absolute LSS	L4–5: grade III
21	Erosive osteochondrosis L5–S1 Modic III° and absolute LSS	L4–5: grade II
22	Degenerative spondylolisthesis I° L4–5 and absolute LSS	L3–4: grade III, relative LSS

### The implant

**Fig. 1:**
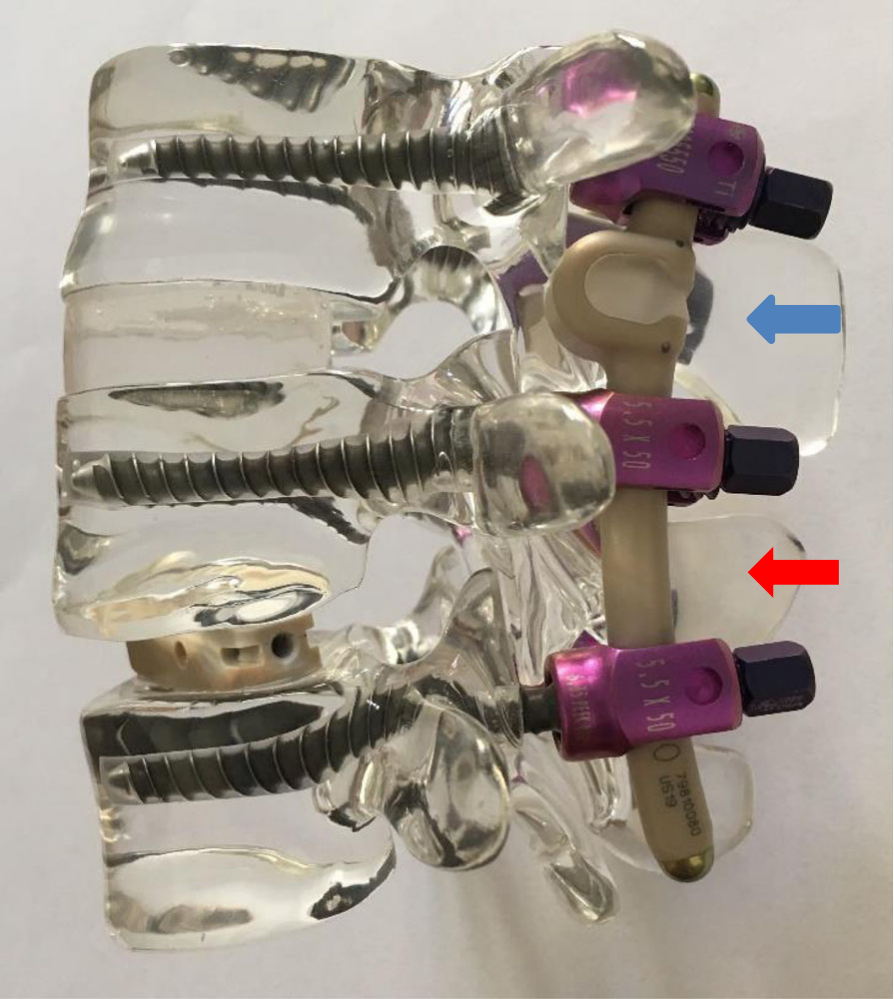
CD Horizon BalanC™. The dynamic section is composed of PEEK and silicon (blue marker), while the fusion section is composed of PEEK (red marker)

The CD Horizon BalanC™ (Medtronic Co., Minneapolis, USA) is a dynamic posterior pedicle screw/rod-based stabilization device, with the dynamic part of the rod constructed of polyether ether ketone (PEEK) and silicone (Ø 6.0; lordotic bend). The fusion portion is entirely made of PEEK (Fig 1). The silicon and PEEK hinge is designed to reduce stress on the adjacent level by restricting extreme ROM. The pedicle screws are made of standard titanium and are comparable to the screws used in the rigidly stabilized level.

### Data collection and outcomes

Patients underwent clinical examinations and radiographs preoperatively and at 1 and 2 years after surgery. Evaluation of the clinical data was based on the German Spine Registry using the Core Outcome Measure Index (COMI) score, the Operation 2011 form, and a visual analogue scale (VAS) for back and leg pain. Data regarding length of hospital stay, operation time, perioperative and postoperative complications, blood loss, and ASA classification were collected by the German Spine Registry. Patients filled out the questionnaires (COMI and VAS) preoperatively and at the 1- and 2-year FU examinations.

The radiological examination contained X-rays of the lumbar spine in anterior–posterior and lateral views taken preoperatively and at 1 and 2 years after surgery. All X-rays were performed in a standing position. Radiological signs of degeneration in the segment adjacent to the fusion (topping off segment) and the segment cranial to the topping off were recorded. Degeneration was categorized according to Weiner’s classification [16], and a segment was classified as degenerated if it achieved a score of two or more.

Weiner’s classification:

Radiographic scoring system for osteoarthritis of the lumbosacral spine:

Intervertebral disc:

0 = no disease. Defined by normal disc height, no spur formation, no eburnation, and no gas present.

1 = mild disease. Defined by < 25% disc-space narrowing, small spur formation, minimal eburnation, and no gas present.

2 = moderate disease. Defined by 25–75% disc-space narrowing, moderate spur formation, moderate eburnation, and no gas present.

3 = advanced disease. Defined by > 75% disc-space narrowing, large spur formation, marked eburnation, and gas present [16].

In addition, the pre- and postoperative sagittal parameters (e.g., the segmental endplate angle of the instrumentation and the topping off segment, lumbar lordosis, pelvic incidence, sacral slope, and pelvic tilt) were also compared.

### Statistical analysis

SPSS (version 25, 76 Chicago, IL, USA) was used to evaluate the data. Descriptive and frequency analyses were used to describe the demographic data, clinical data, and outcomes. The COMI score and the radiological sagittal parameter were analyzed using Student’s *t* test for dependent samples, while the VAS scores for back and leg pain were analyzed using the Wilcoxon test. Line diagrams were used to depict the COMI and VAS scores as well as the radiological parameters, with whiskers indicating standard deviation. All reported *P* values have a two-tailed significance level of alpha = 0.05. No adjustment for multiple testing was performed.

## Results

A total of 22 patients (16 female and 6 male) with symptomatic degenerative disease or spondylolisthesis of the lumbar spine met the inclusion criteria; 20 patients attended the FU examinations at 1 and 2 years after the procedure. The average age of the patients was 57.6 ± 11.5 (range 41–78) years at the time of surgery.

### Clinical data

The average hospitalization time was 11.8 ± 6.5 (range 5–34) days. PLIF was performed in 18 cases, and TLIF was performed in four cases. Decompression by laminotomy and flavectomy was performed in the topping off (dynamic) segment in four cases. Clinical data are presented in Table 3.

**Table 3:** Clinical data

Case	Operation	Age	Sex	BMI (kg/m^2^)	ASA	Operation time (h)	Intraoperative blood loss (mL)
1	PLIF L4–5 with topping off L3–4	56	F	26–30	2	2–3	100–500
2	TLIF L5–S1 right with topping off L4–5 and foraminotomy L5 right	50	F	26–30	2	2–3	100–500
3	PLIF L4–5 with topping off L3–4	65	F	26–30	2	2–3	500–1000
4	PLIF L5–S1 with topping off L4–5	66	M	20–25	2	2–3	500–1000
5	TLIF L5–S1 left with topping off L4–5	48	F	31–35	2	2–3	500–1000
6	PLIF L4–5 with topping off L3–4	56	F	26–30	2	3–4	500–1000
7	PLIF L5–S1 with topping off L4–5	50	F	> 35	2	2–3	100–500
8	PLIF L5–S1 with topping off L4–5	50	M	20–25	2	2–3	100–500
9	PLIF L4–5 with topping off L3–4, flavectomy and laminotomy L3–4 bilateral	72	F	26–30	2	3–4	500–1000
10	TLIF L4–5 left with topping off L3–4, flavectomy and laminotomy L3–4 left	71	F	31–35	2	2–3	1000–2000
11	PLIF L4–5 with topping off L3–4	78	M	< 20	3	1–2	500–1000
12	PLIF L4–5 with topping off L3–4	43	F	> 35	2	2–3	100–500
13	PLIF L4–5 with topping off L3–4	58	M	26–30	2	3–4	500–1000
14	PLIF L4–5 with topping off L3–4, laminotomy and foraminotomy L3–4 bilateral	61	F	20–25	2	2–3	500–1000
15	PLIF L4–5 with topping off L3–4	43	M	< 20	1	2–3	100–500
16	PLIF L5/S1 with topping off L4–5	41	M	31–35	2	2–3	500–1000
17	PLIF L5–S1 with topping off L4–5, laminotomy and flavectomy L3–4 left	64	F	31–35	3	3–4	1000–2000
18	PLIF L5–S1 with topping off L4–5 and sequestrectomy L4–5 left	58	F	< 20	1	2–3	100–500
19	PLIF L4–5 with topping off L3–4	71	F	26–30	3	3–4	1000–2000
20	TLIF L5–S1 right with topping off L4–5	45	F	20–25	2	1–2	100–500
21	PLIF L5–S1 with topping off L4–5	45	F	20–25	2	2–3	1000–2000
22	PLIF L4–5 with topping off L3–4	76	F	> 35	3	3–4	500–1000

### Clinical outcomes

**Fig. 2:**
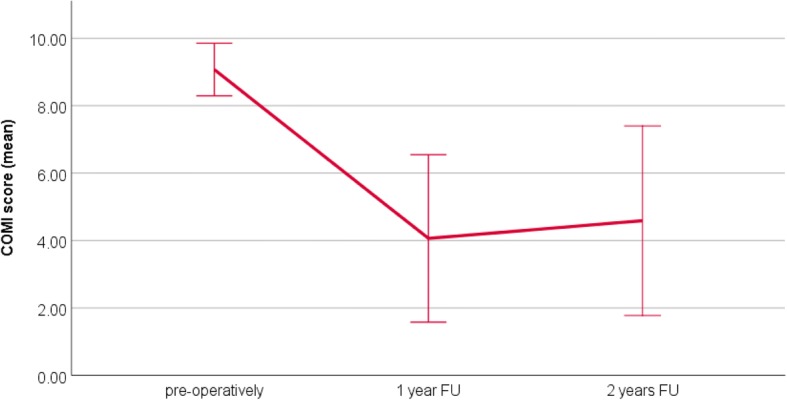
The COMI score preoperatively and at the 1- and 2-year FU. The whiskers indicate the standard deviation. The COMI score is significantly improved at 1 and 2 years after surgery

**Fig. 3:**
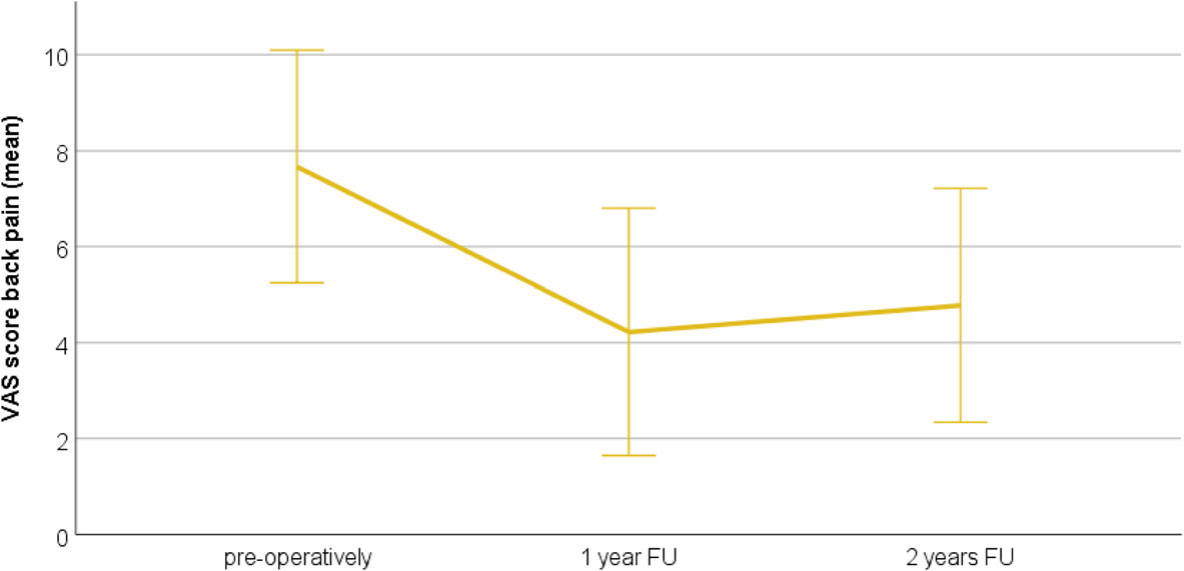
The VAS score for back pain preoperatively and at the 1- and 2-year FU. The whiskers indicate the standard deviation. The VAS score for back pain is significantly improved at 1 and 2 years after surgery

**Fig. 4:**
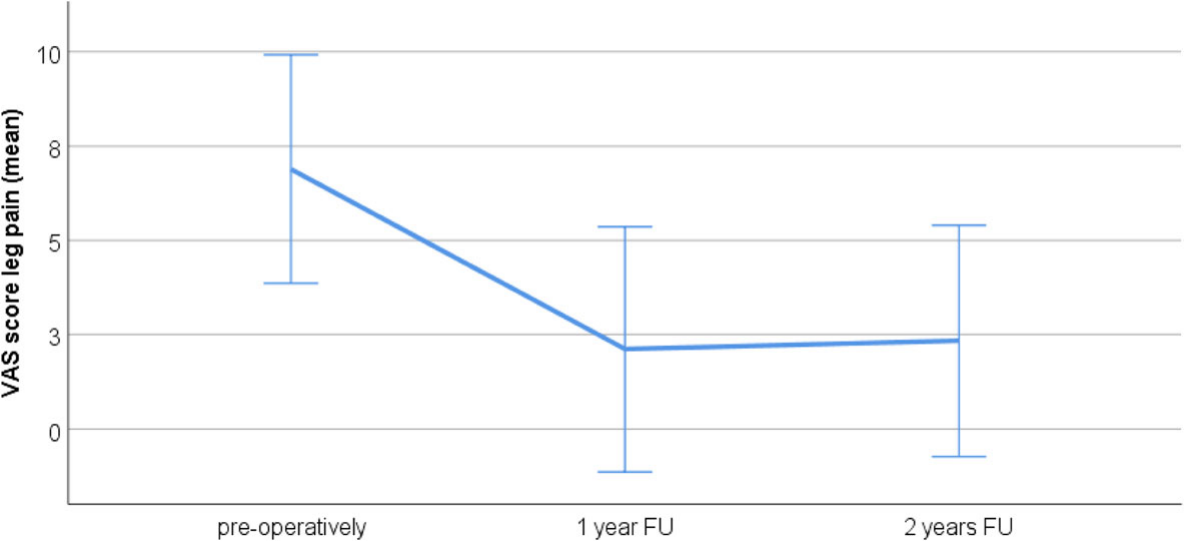
The VAS score for leg pain preoperatively at the 1- and 2-year FU. The whiskers indicate the standard deviation. The VAS score for leg pain is significantly improved at 1 and 2 years after surgery

The average COMI and VAS scores preoperatively and at the 1- and 2-year FU are presented in Table 4. There was a significant reduction in the COMI score at the 1-year (*P* < 0.001) and 2-year (*P* < 0.001) FU compared to preoperatively. VAS scores for both back and leg pain significantly reduced at the 1- and 2-year FU (back pain: *P* = 0.002 at 1 year and *P* = 0.003 at 2 years vs. preoperatively; leg pain: *P* = 0.001 at 1 and 2 years vs. preoperatively). Figs. 2, 3, and 4 illustrate the development of clinical outcomes during the FU.

**Table 4:** Clinical outcomes: mean COMI score, mean VAS score for back pain, and mean VAS score for leg pain preoperatively and at the 1-year and 2-year FU

	Preoperatively	1-year FU	2-year FU
COMI score	9.0 ± 0.9 (range 6.7–10.0)	4.2 ± 2.5 (range 0–7.5)	4.7 ± 2.7 (range 0.2–8.3)
VAS back pain	7.7 ± 2.4 (range 0–10)	4.25 ± 2.4 (range 0–8)	4.7 ± 2.3 (range 0–9)
VAS leg pain	7.1 ± 2.9 (range 0–10)	2.2 ± 3.2 (range 0–8)	2.3 ± 2.35 (range 0–8)

### Radiological outcome

**Fig. 5:**
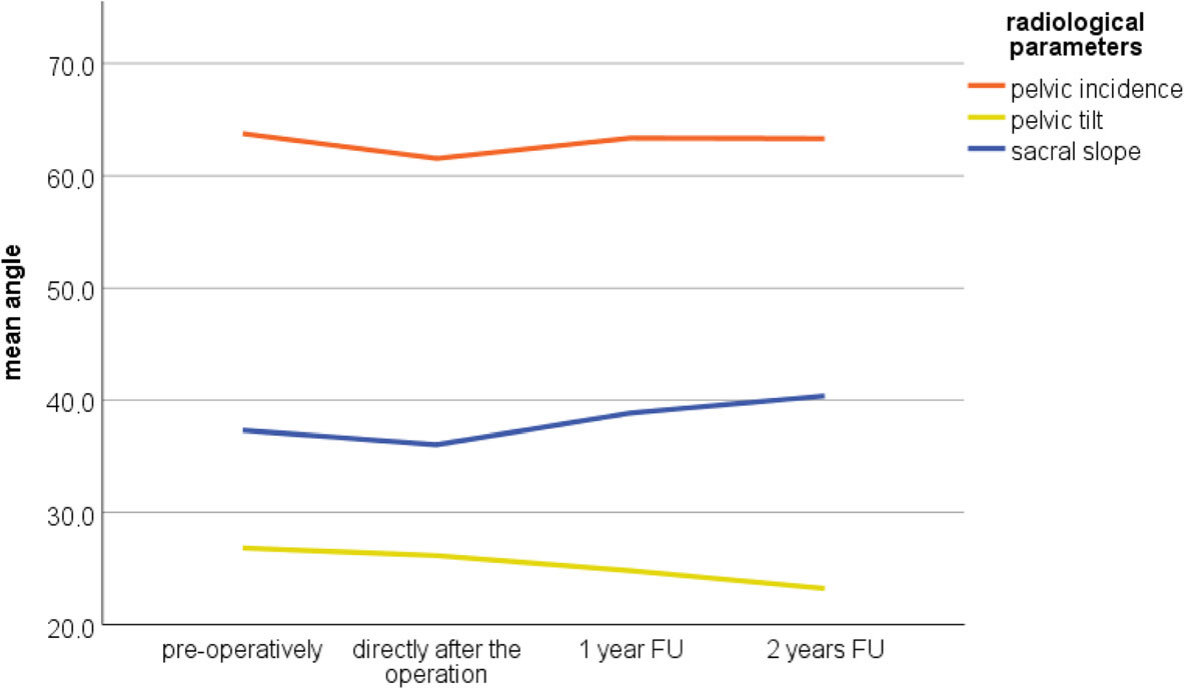
The positive-valued radiological parameters preoperatively, directly after the operation at the 1- and 2-year FU. Pelvic incidence (PI; orange line), pelvic tilt (PT; yellow line), and sacral slope (SS; blue line). The figure shows a slight reduction in the PI after the operation (2.3°); however, this was comparable to the preoperative value at the 1- and 2-year FU

**Fig. 6:**
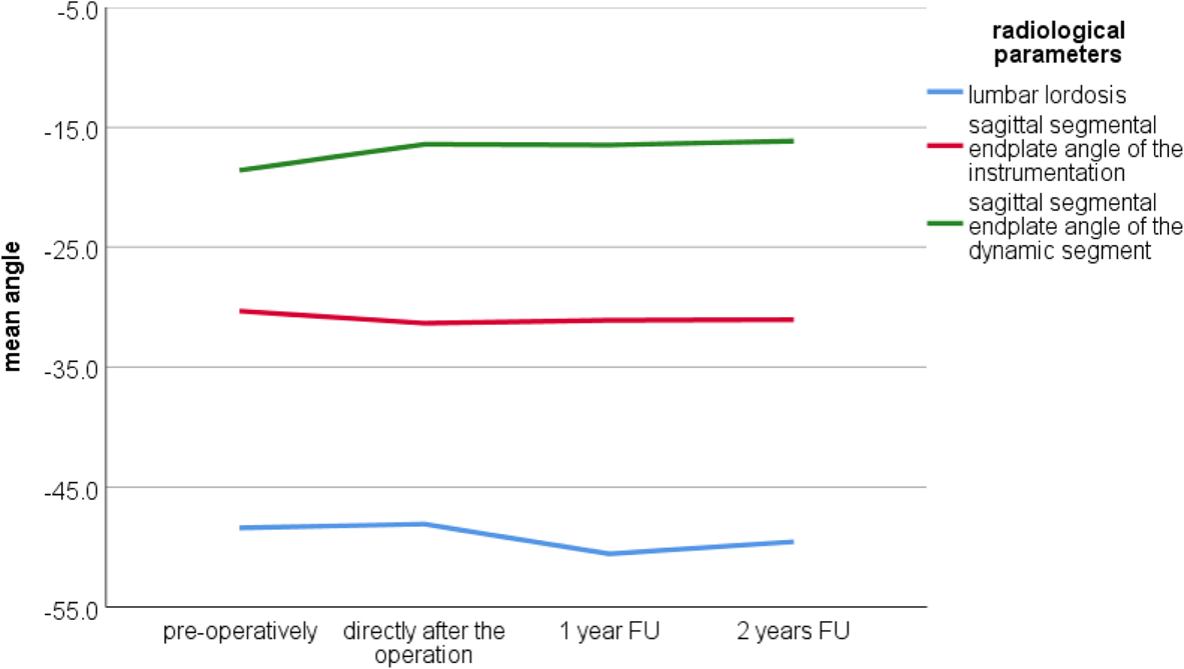
The negative-valued radiological parameters preoperatively, directly after the operation and at the 1- and 2-year FU. Lumbar lordosis (LL; light blue), sagittal segmental endplate angle of the instrumentation (SSEI; purple line), and sagittal segmental endplate angle of the dynamic segment (SSED; green line). The mean SSED was reduced after the operation. The reduction in segmental lordosis remained during the follow-up. Mean SSEI increased slightly after the operation and remained during the follow-up

The detailed radiological results of lumbar lordosis, the sagittal segmental endplate angle of the instrumentation, the sagittal segmental endplate angle of the dynamic segment, pelvic incidence, pelvic tilt, and sacral slope are presented in Table 5. Figs. 5 and 6 illustrate the development of the radiological parameters preoperatively, directly after the operation and during the FU.

**Table 5:** Radiological outcomes: directly after surgery, at 1-year and 2-year FU (* = significant)

	Preoperatively	Directly after surgery	1-year FU	2-year FU
Lumbar lordosis (°)	− 48.4 ± − 13.0 (range − 25.0 to − 77.0)	− 48.0 ± − 9.9 (range − 27.0 to − 73.0) *P* = 0.886	− 50.6 ± − 11.2 (range − 27.0 to − 73.0) *P* = 0.562	− 49.6 ± − 10.4 (range − 30.0 to − 65.0) *P* = 0.835
Pelvic incidence (°)	63.8 ± 11.8 (range 38.0–85.0)	61.5 ± 9.9 (range 38.0–85.0) *P* = 0.141	63.4 ± 7.5 (range 51.0–72.0) *P* = 0.332	63.3 ± 9.5 (range 40.0–76.0) *P* = 0.681
Pelvic tilt (°)	26.8 ± 8.7 (range 10.0–40.0)	26.8 ± 8.7 (range 10.0–40.0) *P* = 0.561	24.8 ± 6.6 (range 9.0–34.0) *P* = 0.018*	23.2 ± 5.9 (range 10.0–35.0) *P* = 0.027*
Sacral slope (°)	37.3 ± 9.6 (range 20.0–56.0)	37.3 ± 9.6 (range 20.0–56.0) *P* = 0.329	38.8 ± 6.3 (range 30.0–52.0) *P* = 0.244	40.4 ± 9.7 (range 23.0–58.0) *P* = 0.060
Sagittal segmental endplate angle of the instrumentation (°)	− 30.3 ± − 10.3 (range − 7.0 to − 50.0)	− 31.3 ± − 5.2 (range − 20.0 to − 40.0) *P* = 0.949	− 31.1 ± − 7.6 (range − 15.0 to − 46.0) *P* = 0.609	− 31.1 ± − 7.0 (range − 12.0 to − 43.0) *P* = 0.622
Sagittal segmental endplate angle of the dynamic segment (°)	− 18.6 ± − 6.8 (range −6.0 to − 35.0)	− 16.4 ± − 5.0 (range − 9.0 to − 30.0) *P* = 0.063	16.5 ± − 6.0 (range − 7.0 to − 30.0) *P* = 0.170	− 16.1 ± − 5.5 (range − 10.0 to − 28.0) *P* = 0.044*

Interestingly, a 2.2° reduction in the mean sagittal segmental endplate angle of the dynamic segment was observed directly after surgery. The segmental kyphosis remained during the FU examinations. A reduction in the segmental lordosis of the dynamic segment could have a negative effect on the sagittal balance of the lumbar spine; however, no influence was observed in the development of lumbar lordosis. This reduction tended to be significant (*P* = 0.063). At the 2-year FU, there was a significant reduction (*P* = 0.044) of the sagittal segmental angle.

### Complications

An incidental durotomy was documented as a perioperative complication in one case. Surgical postoperative complications were reported in three patients. One patient developed a lumbar radiculopathy without a neurological deficit. One patient needed revision surgery because of a misplaced pedicle screw. The pedicle screw misplacement was diagnosed after a computed tomography scan of the lumbar spine was performed due to a persistent radiculopathy without a neurological deficit. One case reported a superficial wound infection. A general complication of pulmonary disease (pneumonia) was reported in one case.

**Fig. 7:**
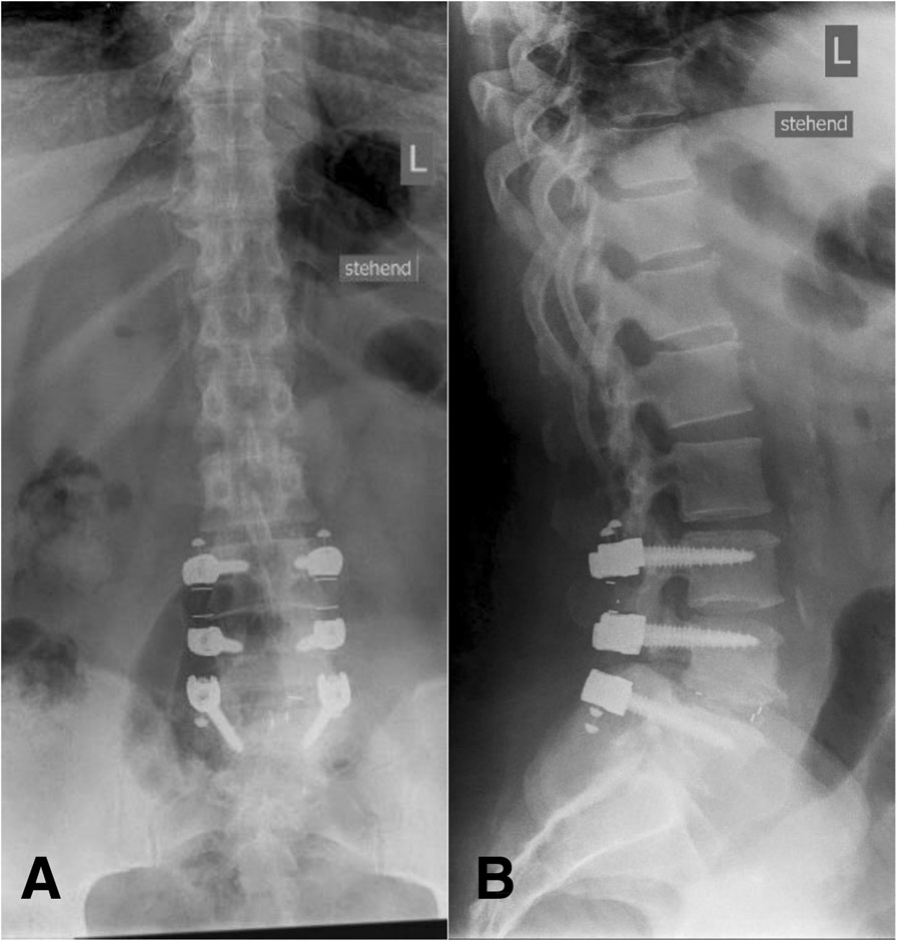
Lumbar spine anterior–posterior (**a**) and lateral (**b**) radiographs immediately after surgery

**Fig. 8:**
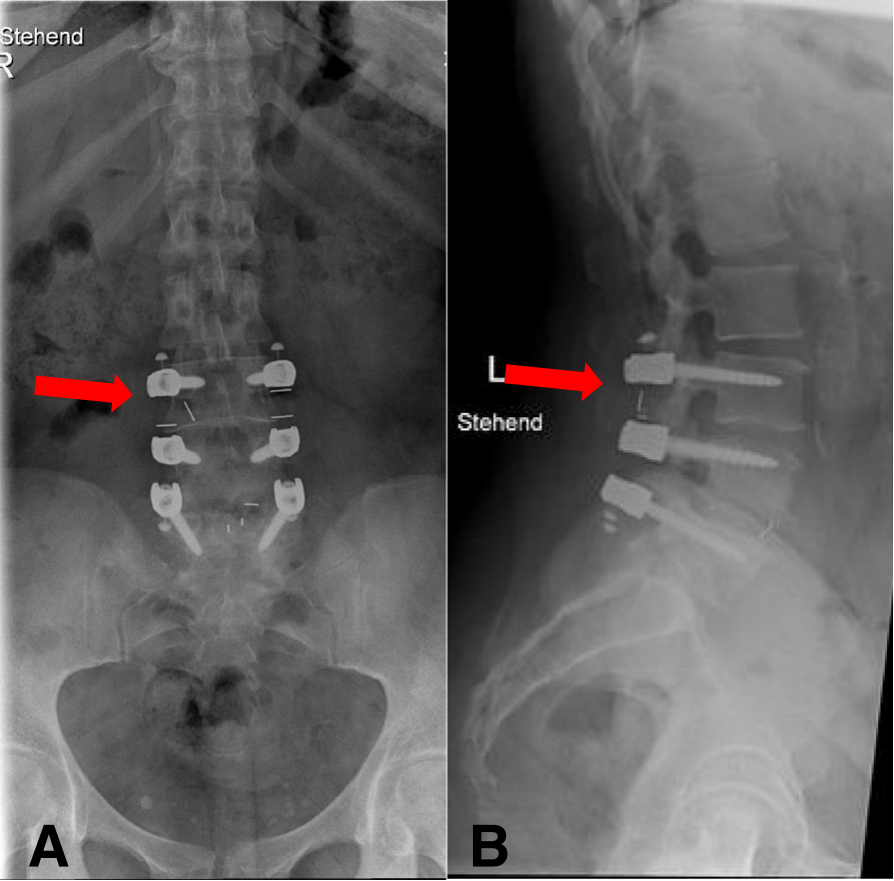
Lumbar spine anterior–posterior (**a**) and lateral (**b**) radiographs at the 2-year FU. The red marker shows breakage of the PEEK and silicone C-shaped dynamic part at segment L3–4

**Fig. 9:**
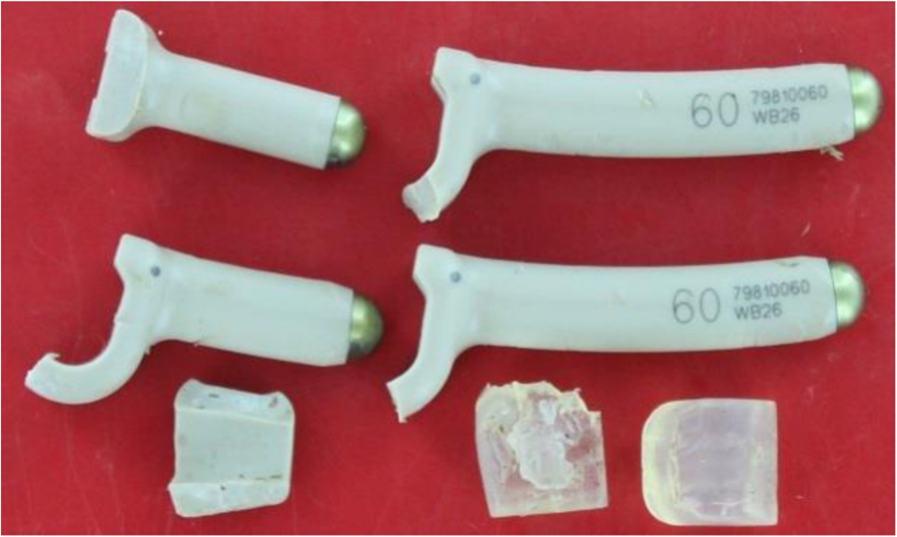
A BalanC rod implant removed from a patient who required surgical revision due to back pain. Broken dynamic (PEEK and silicone) C-shaped part of both rods

Obvious signs of material failure in the dynamic part of the implant were identified in four cases (18%) (Figs. 7 and 8). In one of these cases, revision surgery was necessary due to persisting back pain during the FU visits (Fig. 9). The other three patients with material failure within the dynamic portion of the implant did not require any revision surgery due to a reduction in back pain and a sufficient clinical outcome.

**Fig. 10:**
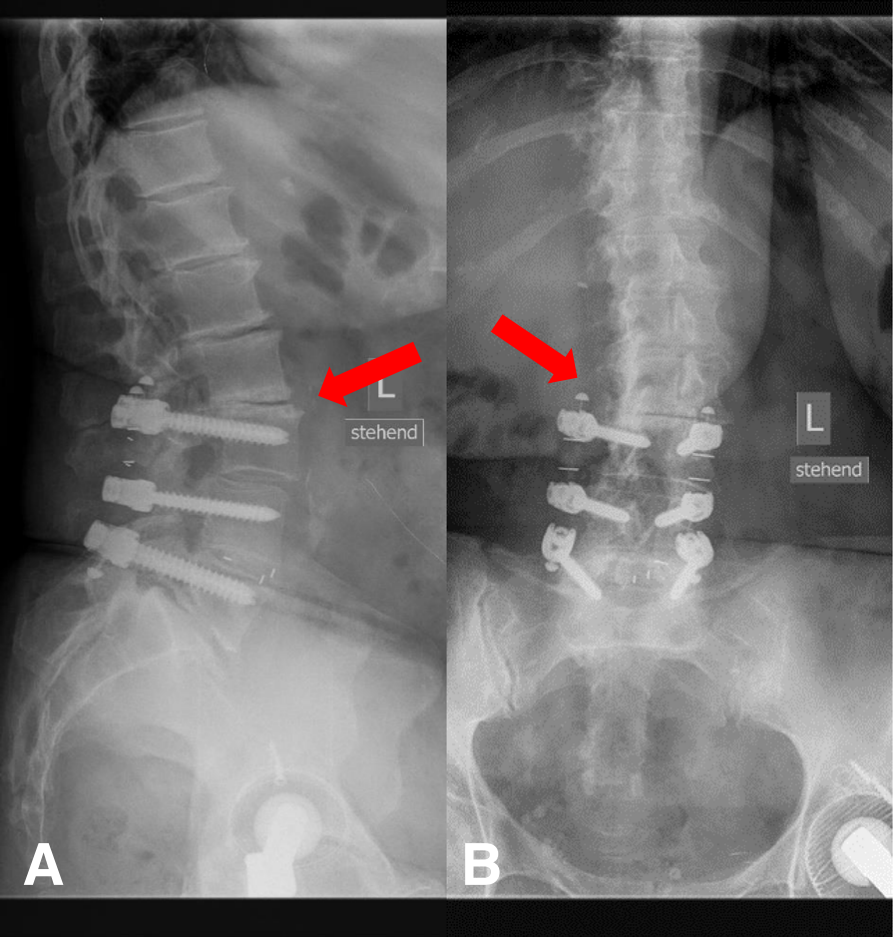
Lumbar spine lateral (**a**) and anterior–posterior (**b**) radiographs at 2 years after surgery. Radiological signs of degeneration (red marker) in the segment cranially adjacent to the topping off segment

In addition, radiological signs of ASD within the segment cranially adjacent to the dynamic instrumented segment were evident in three cases (15%). These cases had a Weiner’s grade of three (Fig. 10). The COMI scores in these three cases at the 2-year FU were 6.2, 1.15, and 1.6. The VAS scores for back pain were 2, 3, and 6, and the VAS scores for leg pain were 0, 0, and 1. No cases of ASD were documented in the topping off segment.

## Discussion

The development of ASDi has prompted the introduction of new operating methods, such as dynamic and semi-rigid implants, for the treatment of degenerative diseases and spondylolisthesis of the lumbar spine. These implants differ in terms of the materials used and the design and biomechanical properties of the dynamic section [12]. Dynamic spine implants allow mobility of the instrumented segment; however, some biomechanical studies have reported small differences in the mechanical performance of posterior dynamic and rigid implants [17].

Many studies reported adequate long-term clinical outcomes after posterior dynamic stabilization of the lumbar spine. In one retrospective study of 299 patients with a mean FU of 9 months, Greiner-Perth et al. achieved good and stable clinical results using the DSS device (Paradigm Spine, LCC, New York, NY, USA) [18]. In a 2-year FU study of 20 patients treated with the Accuflex (Globus Medical, Inc.) posterior dynamic stabilization device, Reyes-Sanchez et al. reported improvements in pain and quality of life [19]. Overall, dynamic posterior stabilization devices appear to provide convincing clinical results [20, 21]. Nonetheless, it remains uncertain whether dynamic posterior stabilization provides better results than traditional lumbar spinal fusion.

In addition, there is no convincing data regarding the hybrid posterior stabilization systems (topping off) during long-term FU [22]. The findings in this study indicate a significant reduction in back and leg pain as well as a significant improvement in clinical outcomes (according to COMI measurement) at 2 years after hybrid lumbar spine stabilization. These clinical results correlate with the outcomes of lumbar fusion and decompression that are summarized in the literature [1, 2].

However, the aim of this study was to examine whether the use of a hybrid lumbar spinal stabilization system and the resulting supplementary instrumentation of a segment offered any benefit to the patient. Hybrid posterior stabilization systems were developed to protect the adjacent segment from hypermobility after lumbar spinal fusion, as hypermobility of the adjacent segment can lead to ASDi [23, 24]. Biomechanical studies examining hybrid dynamic lumbar stabilization reported reduced mobility of the adjacent segment after hybrid dynamic stabilization [11]. However, this precautionary fixation is not far from a two-level fusion [25]. This can lead to hypermobility of the segment adjacent to the dynamic stabilization [11, 26]. Thus, ASD can occur in the next cranial segment, as was reported in this study. In this sense, hybrid posterior stabilization can lead to bisegmental fusion and patients underwent supplementary iatrogen operation morbidity.

One possible advantage of hybrid fusion versus single-level fusion is the option to perform extended decompression of the neural structures in the cranial segment and protect the segment from instability [27]. A long-term clinical study comparing single-level lumbar spinal fusion with hybrid lumbar spinal instrumentation identified no clinical benefits of hybrid fusion. In a prospective study comparing single-level fusion and hybrid instrumentation in the lumbar spine, Putzier et al. followed patients for 6 years and reported comparable functional outcomes. These authors used the Allospine Dynesys Transition device (Zimmer, Winterthur, Switzerland) [22]; however, biomechanical studies using Dynesys reported increased stiffness applied to the adjacent segment. Thus, Dynesys performs comparably to rigid implants [28, 29]. In a retrospective study, Baioni et al. assessed clinical and radiological outcomes after hybrid lumbar spinal fusion using the Dynesys implant. They reported satisfying clinical outcomes at the 5-year FU. In addition, the prevalence of radiographic ASD was 10% (3/30 patients) [30]. In contrast, the results reported in our study show a radiographically detectable ASD rate of 15% within the segment superior to the dynamic instrumented level and, interestingly, no radiological or clinical signs of ASD or ASDi within the dynamic instrumented level. The prevalence of ASD in the segment cranially adjacent to the dynamic instrumented level (15%) correlates with the incidence of ASD after lumbar spine fusion reported in the literature [8, 9]. In this respect, the results show that hybrid lumbar spinal fusion using the CD Horizon BalanC™ rod system is not able to prevent the development of ASD. One possible reason for the development of ASD in the segment cranial to the dynamic instrumentation could be the reduction of lordosis of the dynamic instrumented segment affecting the sagittal balance of the lumbar spine. In our study, we reported a mean reduction of the sagittal endplate angle of the dynamic instrumentation of 2.2.

In a retrospective study of 24 patients (mean FU of 8 months), Maserati et al. assessed clinical and radiological outcomes after hybrid lumbar instrumentation with the DTO. They found improvements in pain and symptomatic degeneration at the dynamically stabilized segment in one case and above the dynamically stabilized segment in two cases [31].

A biomechanical study examining the performance of the dynamic part of the CD Horizon BalanC™ rod reported stiffness in the ROM (except for axial rotation) that was similar to that seen with rigid implants [25]. According to the radiological findings reported in this study, this biomechanical study supports the hypothesis that load sharing is transferred to one level above the instrumented segments. To the author’s knowledge, there has been no published data regarding clinical outcomes after hybrid lumbar instrumentation with the CD Horizon BalanC™ rod system. The dynamic part of the CD Horizon BalanC™ rod is constructed of PEEK and silicone, making it different from the DTO. The results reported here show that there were significant improvements with regard to back and leg pain 2 years after surgery.

The prevalence of material failure in the dynamic implant section was 18% in this study. In these cases, the adjacent segment was no longer protected by the topping off part of the implant. In the author’s opinion, this high prevalence indicates a weak area of the implant. Material failure in other dynamic implants has also been reported [19, 32]; for example, Hoff et al. performed a prospective study over a 24-month period to compare single-level dynamic and hybrid instrumentation with the CD Horizon Agile spinal system (Medtronic, Memphis, TN, USA) and reported not only satisfactory functional outcomes but also a high failure rate of the dynamic portion of the implants [32]. Reports of material failure in dynamic spine implants raise concerns about the use of these devices. In addition, the reliability of before-market implant tests is questionable.

### Limitations

First, there was no control group, and due to the fact that the data were obtained from patients who underwent surgery in a single center, there may also have been selection bias. Therefore, a further randomized controlled study should be conducted in multiple hospitals. Second, pathoanatomic risk factors (e.g., facet tropism and sagittalization and horizontalization of the lamina) were not observed.

## Conclusion

The high rate of material failure (18%) and the onset of adjacent segment alteration superior to the dynamic instrumented level (15%) suggests that the use of a “topping off” device is not able to reduce the incidence of ASD, whereas the reported pain decrease in the study correlates with the outcomes of lumbar fusion and decompression summarized in the literature [1, 2]. Furthermore, the implant is obsolete due to the high failure rate. In this aspect, we conclude that supplementary dynamic instrumentation of the segment cranial to the rigid instrumentation does not offer any benefits to the patient.

## Abbreviations

ASA: American Society of Anesthesiologists
ASD: Adjacent segment degeneration
ASDi: Adjacent segment disease
COMI: Core outcome measure index
FU: Follow-up
LSS: Lumbar spinal stenosis
MRI: Magnetic resonance imaging
PEEK: Polyether ether ketone
PLIF: Posterior lumbar interbody fusion
ROM: Range of motion
TLIF: Transforaminal lumbar interbody fusion
VAS: Visual analogue scale
